# A Phase II Trial of Stereotactic Ablative Body Radiotherapy for Low-Risk Prostate Cancer Using a Non-Robotic Linear Accelerator and Real-Time Target Tracking: Report of Toxicity, Quality of Life, and Disease Control Outcomes with 5-Year Minimum Follow-Up

**DOI:** 10.3389/fonc.2014.00279

**Published:** 2014-11-14

**Authors:** Constantine Mantz

**Affiliations:** ^1^21st Century Oncology, Fort Myers, FL, USA

**Keywords:** prostate cancer, SABR, hypofractionation

## Abstract

**Purpose/Objective(s):** Herein, we report the results of an IRB-approved phase II trial of Varian Trilogy/TrueBeam-based stereotactic ablative body radiotherapy (SABR) monotherapy for low-risk prostate cancer using the Calypso^®^ System to provide real-time electromagnetic tracking of the prostate’s position during treatment delivery.

**Materials/Methods:** A total of 102 low-risk patients completed protocol treatment between January 2007 and May 2009. A total dose of 40.0 Gy in 5 every-other-day fractions of 8.0 Gy was prescribed to the planning target volume. Target setup and tracking procedures were as follows: (1) the Calypso^®^ System was used to achieve target setup prior to each fraction; (2) conebeam CT imaging was then used for correction of setup error and for assessment of target and organs-at-risk deformations; (3) after treatment delivery was initiated, the Calypso^®^ System then provided real-time intrafractional target tracking. The NCI CTCAE v3.0 was used to assess urinary and rectal toxicity during treatment and at defined follow-up time points. Biochemical response and quality of life measurements were made at concurrent follow-up points.

**Results:** Urinary toxicities were most common. At 6 months, 19.6, 2.9, and 4.9% of patients reported grades 1–2 urinary frequency, dysuria, and retention, respectively. Rectal toxicities were uncommon. By 12 months, 2.9% of patients reported painless rectal bleeding with subsequent symptom resolution without requiring invasive interventions. Quality of life measurements demonstrated a significant decline over baseline in urinary irritative/obstructive scores at 1 month following SABR but otherwise did not demonstrate any difference for bowel, bladder, and sexual function scores at any other follow-up time point. One patient suffered biochemical recurrence at 6 years following SABR.

**Conclusion:** At 5 years, minimum follow-up for this favorable patient cohort, prostate SABR resulted in favorable toxicity, quality of life, and biochemical outcomes.

## Introduction

Stereotactic ablative body radiotherapy (SABR) is a recent external beam radiation treatment modality in the curative management of localized prostate cancer. What primarily distinguishes SABR from other external beam therapies is its brief treatment schedule of five or fewer treatment fractions to deliver a biologically effective radiation dose to the prostate gland. Improvements in linear accelerator targeting and beam delivery performance have allowed for the consideration of safely compressing conventional treatment courses of 7–9 weeks into more abbreviated treatment schedules. In 2013, the American Society for Radiation Oncology (ASTRO) endorsed SABR as an appropriate alternative to other, more conventional therapies for selected low- and intermediate-risk disease patients on the basis of published clinical data supportive of its efficacy and safety (1).

As an extremely hypofractionated therapy, SABR tests a radiobiologic hypothesis that promotes fewer and larger fractions for effective prostate cancer irradiation. Dose–response analyses of disease outcomes have generally concluded that the α:β ratio for prostate cancer may be exceptionally low, with studies reporting estimated ratios as low as 1.5 (2–9). Caveats to this conclusion of a low α:β ratio for prostate cancer include (1) the mathematical assumptions made in some studies in order to allow for comparisons between external beam radiotherapy and brachytherapy outcomes and (2) the lack of certainty that the linear-quadratic model may be applied to estimating dose–response when very large fractional doses are considered (10–12). If this conclusion is, however, true, then prostate tumor effect may be highly sensitive to fraction size and perhaps even more so than the late effects of surrounding normal tissue. Therefore, the clinical implication of a hypofractionated treatment schedule for prostate cancer may be improved tumor control rates while maintaining a similar biologically effective dose (BED) for normal tissue late effects when compared to conventionally fractionated regimens.

A fundamental technical demand of a prostate SABR delivery system is the ability to respond to organ motion. Prostate movement is stochastic, meaning that the gland may move without predictability in any direction and at any time (13, 14). In HDR brachytherapy, organ motion is accounted for by intraprostatic placement of afterloading catheters which then move with the gland. For external beam modalities, implanted fiducial markers are commonly used to correct for interfractional organ motion prior to treatment delivery through the use of on-board imaging devices integrated into the linear accelerator platform. However, this may be inadequate for hypofractionated therapy as intrafractional prostate motion may occur with displacements of up to 1 cm (15–20). When a small number of large fractions are administered, there is increased need to account for this source of localization error in order to maintain the minimal PTV margins necessary for SBRT and, consequently, reduce normal tissue complication probability and increase tumor control probability (21, 22). An optimal solution for external beam modalities is therefore a system that couples real-time tracking of the prostate to the linear accelerator and permits immediate corrective action to changes in prostate position within a treatment fraction.

Varian Trilogy and TrueBeam (Varian Medical Systems, Inc., Palo Alto, CA, USA) are rapid output linear accelerators with an integrated image guidance system. Briefly, each linear accelerator’s platform provides image guidance by robotically extending an opposed kilovolt x-ray source and amorphous silicon flat panel imager tandem over the target volume. The source-imager tandem then acquires planar or volumetric imaging of implanted fiducial markers and/or the target to permit corrections of target setup error prior to treatment. During treatment, the beam may be stopped and imaging repeated to identify and correct intrafractional target motion. While technically possible, repeat imaging during a treatment fraction is time-consuming and is still unlikely to identify every instance of target excursion outside the treatment volume. The Calypso^®^ System (Varian Medical Systems, Inc., Palo Alto, CA) is a real-time target tracking system that provides a more efficient solution to the problem of intrafractional target motion. This system consists of an electromagnetic array that can detect the positions of signal-emitting transponders implanted in the target and provides continuous information of target position with sub-millimeter accuracy.

This report describes toxicity, quality of life, and biochemical disease outcomes from an IRB-approved, phase II trial of conventional linear accelerator-based SABR monotherapy in the treatment of low-risk prostate cancer using the Calypso^®^ System for intrafractional real-time target tracking.

## Materials and Methods

### Patient selection

A total of 102 patients were treated from January 2007 to May 2009. All of the following eligibility criteria were required for enrollment: clinical stage T1c–T2a, presenting serum PSA ≤10 ng/ml, and GS ≤6. Patients with GS 7 were also eligible for enrollment if the primary histologic score were three and if ≤25% of biopsy cores were positive. Patients were not eligible if any of the following medical factors were present: prostate US volume >60 cc, prior hormonal therapy, international prostate symptom score (IPSS) >18, history of TURP, history of colostomy, history of pelvic radiotherapy, or history of chemotherapy. Technical ineligibility factors included implanted metallic hip prosthetic devices and >21 cm distance between the electromagnetic array and implanted transponders, either of which could compromise targeting and tracking performance of the Calypso^®^ System.

### Protocol treatment planning

Patients underwent transponder implantation at least 5 days before treatment planning imaging to allow for resolution of post-implant edema. Three transponders were placed transrectally under ultrasound guidance and distributed such that at least 1.5 cm spacing was achieved among them.

CT imaging was performed for volume delineation and dose calculation. Supine patient immobilization was achieved through use of a Vac-Lok device (Medtec, Inc., Orange City, IA, USA) placed under the lower torso, pelvis, and thighs. The CT study set was acquired with 1.25-mm slice thickness and without contrast.

The CTV consisted of the prostate only. The PTV was created by a uniform 2-mm expansion of the CTV in all dimensions. The rectum was contoured as a solid structure including all intraluminal contents from the sigmoid flexure to the ischial tuberosities, encompassing a length of roughly 15 cm. The bladder was also contoured as a solid structure inclusive of all contents from the dome to the bladder neck. The femoral heads were contoured from the level of the acetabula to just inferior to the greater trochanters. The penile bulb was contoured electively for potent patients from its origin just inferior to the urogenital diaphragm and then anteriorly for 2 cm toward the corpora cavernosa.

A total dose of 40.0 Gy in five fractions was prescribed to the PTV. This schedule was selected such that its late effects BED would be similar to that for 86.4 Gy in 48 fractions – a conventionally fractionated, dose-escalated schedule previously reported with low rates of late genitourinary and gastrointestinal toxicities (23). Assuming both a late effects α:β of 3 and a 5% increment to the conventional fractionation late effects BED for calculating the SABR late effects BED (this increment assumed on the basis of improved intrafractional target localization with the Calypso^®^ System), we may then use the BED formalism to calculate an equivalent SABR dose schedule:

$$\begin{array}{llll} \mathrm{BED} & =\left(\mathrm{nd}\right)+\left(\frac{nd^{2}}{\alpha \text{/}\beta }\right)\; & & \;\\ \mathrm{BE}D_{\mathrm{conv late effects}} & =\left(\mathrm{48}\cdot 1\mathrm{.8}\right)+\left(\frac{48\cdot 1.8^{2}}{3}\right)=138.24\; & & \;\\ \mathrm{BE}D_{\mathrm{SABR late effects}} & =138.24+\left(138.24\cdot 5\%\right)=145.15\; & & \;\\ 145.15 & =\left(5\cdot d_{\mathrm{SABR}}\right)+\left(\frac{5\cdot {\left(d_{\mathrm{SABR}}\right)}^{2}}{3}\right)\; & & \;\\ d_{\mathrm{SABR}} & ≅8.0\;\mathrm{Gy}\; & & \; \end{array}$$

**Figure 1:**
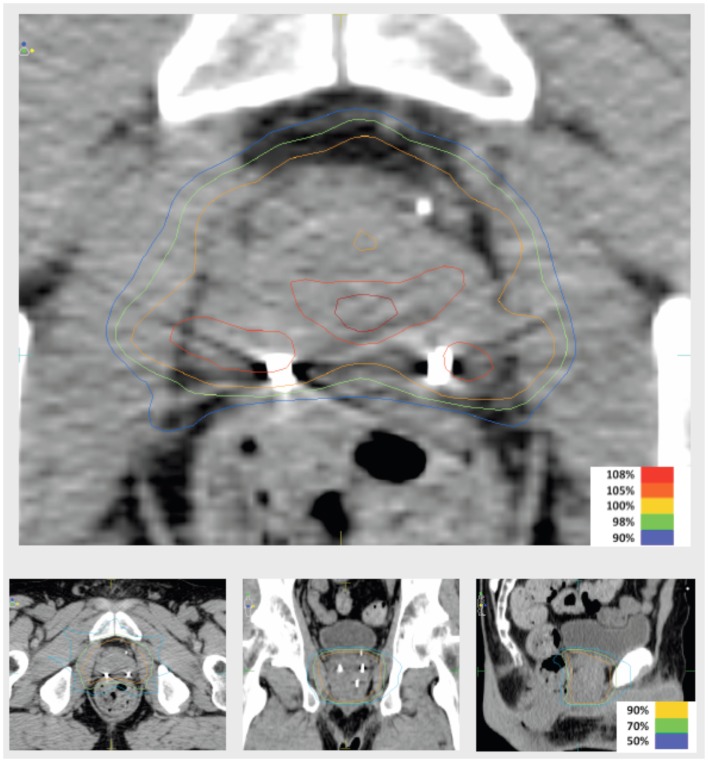
**Representative isodose distributions of an SABR treatment plan**.

**Figure 2:**
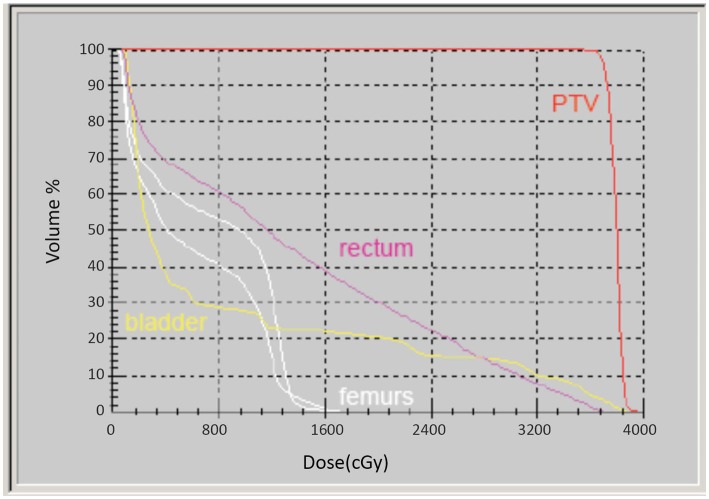
**Representative dose-volume histogram of an SABR treatment plan**.

Intensity-modulated treatment planning was performed using CMS XiO Planning System (CMS Software, Inc.; Elekta Group; Stockholm, Sweden). A typical treatment plan consisted of seven or nine intensity-modulated fields arranged isocentrically in a non-opposing, coplanar orientation. Each field consisted of 10 intensity levels. Target volume coverage acceptance parameters included PTV_40Gy_ ≥98% and CTV_40Gy_ ≥100%. Organs-at-risk (OAR) dose-volume constraints for SABR were appropriated from conventional fractionation limits and converted for hypofractionated therapy using BED calculations as above. Required OAR constraints for protocol treatment included rectum D_15_ <32.7 Gy, bladder D_15_ <34.9 Gy, and penile bulb mean dose <22.9 Gy. Representative isodose distributions and dose-volume histograms are provided in Figures 1 and 2.

### Protocol treatment

Treatment was delivered according to an every-other-day schedule over 10 calendar days. Patient preparation consisted of over-the-counter laxatives the evening prior to treatment and then NPO after midnight. This preparation regimen was also used for planning imaging. With Calypso^®^ tracking, an excursion threshold of 2 mm in all dimensions (lateral, anteroposterior, and superoinferior) was assigned. If the Calypso^®^ System detected any target movement beyond this limit, then the treatment beam was immediately stopped and then resumed once the transponder coordinates were detected within the assigned threshold. If the beam remained interrupted for more than two continuous minutes, then the patient and target were realigned before completing treatment.

### Assessments

The NCI CTCAE v3.0 was used to assess urinary and rectal toxicity at baseline, during treatment and at the following post-treatment points: 1, 3, 6, 9, 12, 18, 24, 36, 48, and 60 months. Toxicity outcomes are represented as crude rates at the defined time points. A description of the NCI urinary and rectal toxicity scales is provided in Table 1. Biochemical response determinations were made at concurrent follow-up points. Biochemical failure was defined as post-therapy PSA nadir +2 ng/ml. All patients completed an expanded prostate cancer index composite (EPIC-26) questionnaire prior to treatment and again at 6, 12, 18, 24, 36, 48, and 60 months post-treatment.

**Table 1:** **National Cancer Institute common terminology criteria for adverse events, version 3.0 for (A) urinary and (B) rectal toxicities**.

Adverse event	Grade				
	1	2	3	4	5
**(A) URINARY TOXICITIES**					
Dysuria/hematuria	Asymptomatic	Frequency with dysuria; macroscopic hematuria	Transfusion; IV pain medications; bladder irrigation indicated	Catastrophic bleeding; major non-elective intervention indicated	Death
Incontinence	Occasional; pads not indicated	Spontaneous, pads indicated	Intervention indicated	Operative intervention indicated	–
Stricture/stenosis	Asymptomatic, radiographic or endoscopic findings only	Symptomatic but no hydronephrosis, sepsis or renal dysfunction; dilation or endoscopic repair or stent placement indicated	Symptomatic and altered organ function; operative intervention indicated	Life-threatening consequences; organ failure or operative intervention requiring organ resection indicated	Death
Urinary frequency/urgency	Increase in frequency or nocturia up to 2×normal; enuresis	Increase >2×normal but <hourly	≥1×/h; urgency; catheter indicated	–	–
Urinary obstruction/retention	Hesitancy or dribbling, no significant residual urine; retention occurring during the immediate postoperative period	Hesitancy requiring medication; or operative bladder atony requiring indwelling catheter for <6 weeks	More than daily catheterization indicated; urological intervention indicated	Life-threatening consequences; organ failure; operative intervention requiring organ resection indicated	Death
**(B) RECTAL TOXICITIES**					
Diarrhea	Increase of <4 stools per day over baseline; mild increase in ostomy output compared to baseline	Increase of 4–6 stools per day over baseline; IV fluids indicated <24 hrs; moderate increase in ostomy output compared to baseline	Increase of ≥7 stools per day over baseline; incontinence; IV fluids ≥24 hrs; hospitalization; severe increase in ostomy output compared to baseline	Life-threatening consequences	Death
Hematochezia	Asymptomatic	Symptomatic; banding or medical intervention indicated	Interventional radiology, endoscopic, or operative intervention indicated	Life-threatening consequences	Death
Incontinence	Occasional use of pads required	Daily use of pads required	Interfering with ADL; operative intervention indicated	Permanent bowel diversion indicated	Death
Proctitis	Rectal discomfort, intervention not indicated	Medical intervention indicated	Stool incontinence; operative intervention indicated	Life-threatening consequences	Death

## Results

A total of 102 patients have been treated and followed for a minimum of 5 years. Seventy patients were diagnosed with Gleason score 3 + 3 disease, and 32 patients were diagnosed with Gleason score 3 + 4 disease. Mean PSA at presentation was 7.30 ng/ml (range, 3.42–10.0 ng/ml) for the entire study cohort.

The most commonly observed toxicity was urinary frequency. At 1 month, grades 1–2 urinary frequency were reported by 32.3% of patients with a rate of 19.6% observed at 6 months and subsequent declines thereafter. Two patients experienced grade 3 urinary frequency (frequency greater than every hour) during treatment but did not require catheterization. Grades 1–2 urinary dysuria and retention were reported by 16.6 and 7.8% of patients at 1 month and 2.9 and 4.9% at 6 months, respectively. No grade 3 or greater dysuria or retention was observed. Rectal toxicity was uncommon. At 6 months, two patients reported grade 1 rectal bleeding, and additional patient reported grade 1 bleeding at 12 months. All three patients experienced resolution of their bleeding without intervention. No patient experienced any grade 3 or greater rectal toxicity at any post-SABR follow-up point.

**Figure 3:**
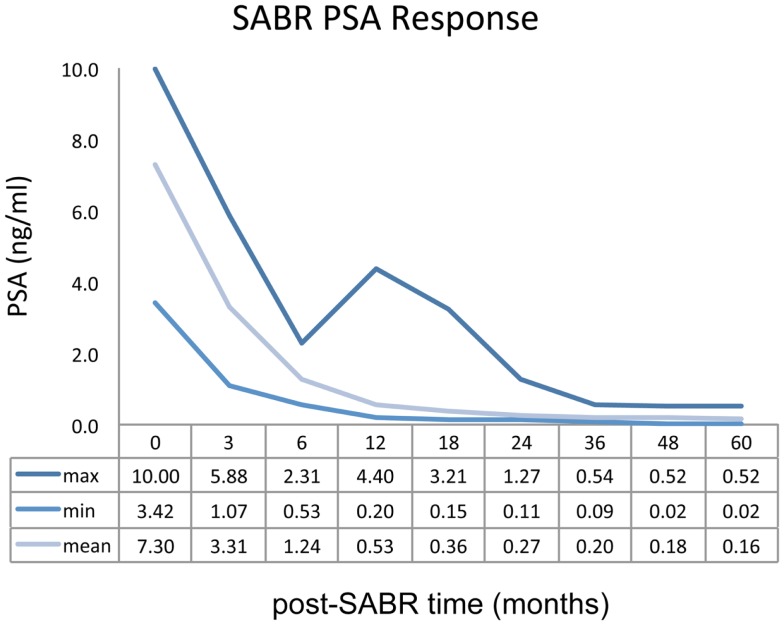
**PSA as a function of post-treatment time**.

Mean PSA measurement for the entire study cohort demonstrated a rapid decline over the first 12 months of follow-up. Biochemical response as a function of post-treatment time is represented graphically in Figure 3. Fifteen patients (14.7%) demonstrated a PSA “bounce” between 12 and 24 months post-SABR, accounting for the sustained elevation observed in the maximum PSA curve. No biochemical failures were noted by 5 years follow-up, although one patient has demonstrated biochemical failure at 6 years.

Quality of life results are presented in Table 2. The EPIC domains studied include urinary incontinence, urinary irritation or obstruction, rectal function, and sexual function. Declines in all domains were observed at 1 month post-treatment, but only urinary irritation/obstruction declined significantly. By 12 months, EPIC domain scores were observed to return to near-baseline levels. By 60 months, scores were observed to decline nominally for all domains but not significantly versus pre-treatment scores.

**Table 2:** **Pre- and post-treatment EPIC scores**.

EPIC domain	Mean EPIC score (SD)				Clinically significant decline ( >0.5 SD)		
	Pre-SABR	Post-SABR at 1 month	Post-SABR at 12 months	Post-SABR at 60 months	At 1 month	At 12 months	At 60 months
Bowel/rectal	92.1 (19.7)	84.4 (17.7)	91.4 (15.1)	91.0 (14.8)	No	No	No
Urinary irritation/obstruction	85.4 (18.3)	74.9 (20.6)	83.2 (16.7)	81 1 (18 9)	Yes	No	No
Urinary incontinence	94.0 (11.9)	90.3 (21.3)	93.1 (16.9)	92 2 (16 7)	No	No	No
Sexual function	51.4 (32.9)	51.3 (25.9)	51.0 (26.0)	47.9 (29.2)	No	No	No

## Discussion

Published clinical evidence generally supports the safety and efficacy of hypofractionation for low-risk prostate cancer. However, many reported studies have included patients across multiple disease risk groups and have variably used androgen deprivation therapy, creating challenges in the interpretation of their findings. Kupelian et al. conducted a phase II trial of external beam radiotherapy delivering 70.0 Gy in 2.5-Gy daily fractions at Cleveland Clinic and reported late grade 3 rectal and urinary toxicities at 5 years of 3 and 1%, respectively, and >90% biochemical disease control at 5 years for low- and intermediate-risk disease patients, some of whom also received androgen suppression therapy (24, 25). RTOG 0415 subsequently randomized low-risk (clinical stage T1-2, PSA <10 ng/ml and Gleason score ≤6) patients either to the Cleveland Clinic hypofractionation scheme or to conventional fractionation (73.8 Gy in 1.8-Gy daily fractions). Patients in either study arm received no hormonal therapy. Published results from this trial are pending.

Other randomized studies of patients in multiple risk categories and treated variably with hormonal therapy have compared hypofractionated treatment schedules to dose-escalated, conventionally fractionated external beam therapy and have generally reported disease control and toxicity equivalence between the two fractionation strategies. The conventional versus hypofractionated high-dose intensity-modulated radiotherapy for prostate cancer (CHHiP) trial enrolled 444 patients with Gleason score ≤7 disease to conventionally fractionated (74 Gy in 2.0-Gy fractions) or hypofractionated (57–60 Gy in 3.0-Gy fractions) radiotherapy (26). No statistically significant differences in urinary or rectal toxicities have been observed between the two arms. An Italian randomized study of 168 high-risk disease patients reported similar late toxicities between its two arms of conventional hypofractionated radiotherapy and statistically significant improvement of biochemical disease control at 3 years for hypofractionated therapy (27). Pollack et al. reported the results of a randomized study of 303 patients with low-to-high-risk localized disease treated with either 76.0 Gy in 2.0-Gy fractions or 70.2 Gy in 2.7-Gy fractions. While disease control outcomes were similar between the two arms, urinary toxicity among patients with AUA scores >10 at baseline was significantly greater for hypofractionated therapy. Otherwise, late toxicities were found to be similar between the two groups (28). M. D. Anderson reported early results of a randomized trial comparing more moderate hypofractionation (72.0 Gy in 2.4-Gy fractions) to conventionally fractionated therapy among 203 patients with low- and intermediate-risk disease. A non-significant trend for increased late rectal toxicity was discovered among hypofractionated patients, particularly for treatment plans demonstrating high doses delivered to >20% of the rectal volume (29).

Several single-institution published prospective studies of SABR fractionation for prostate cancer have generally established the feasibility of delivering very large fraction sizes safely and effectively using modern linac platforms, predominantly the CyberKnife system (30–35). These studies employed treatment schedules of 6.7–10 Gy fractions to total doses of 33.5–50.0 Gy over five fractions. A pooled analysis of published and previously unpublished data was recently reported by King et al. and consisted of 1100 low-to-high-risk disease patients largely treated to a total dose of 36.25 Gy using the CyberKnife system. Some patients also received androgen suppression. For 135 patients with minimum 5-year follow-up, biochemical relapse-free survival was 99 and 93% for low- and intermediate-risk patients, respectively (36). Quality of life outcomes were reported in a separate report by the same group. With 194 patients evaluable at 5 years and using EPIC methodology, declines in urinary and bowel domain scores were observed within the first 3 months post-treatment followed by a return to baseline after 6 months post-treatment. Sexual domain scores were found to have declined predominantly over the first 9 months post-treatment (37). At present, several clinical trials are comparing stereotactic radiotherapy to other fractionation schemes: RTOG 0938 has randomized favorable risk patients to 36.25 Gy in five fractions versus 51.6 Gy in 12 fractions; the PACE trial (prostate advanced in comparative evidence) has randomized low- and intermediate-risk patients to 36.25 Gy in five fractions or 38.0 Gy in four fractions versus laparoscopic prostatectomy or conventionally fractionation radiotherapy; and the HYPO-RT-PC trial (hypofractionated radiotherapy of intermediate-risk localized prostate cancer) is randomizing patients between 42.7 Gy in 7 fractions versus 78.0 Gy in 39 fractions. Results from these studies are pending.

Complementing the pooled analyses of the CyberKnife experience discussed above, this report provides long-term disease control, toxicity, and quality of life outcomes for a patient cohort treated with a modern, non-robotic linear accelerator at a single institution. Broad comparisons of these outcomes between the two reports suggest therapeutic equivalence between the two technology platforms in delivering SABR for localized prostate cancer. Some of the key technical differences between the two platforms merit mention. The SABR platform in this study allows for real-time target tracking with an integrated Calypso^®^ System and is therefore distinguished from the CyberKnife, which instead employs an orthogonal planar x-ray imaging system to obtain non-continuous, three-dimensional positional data of implanted fiducial markers at defined time points during treatment delivery. The Calypso^®^ System generates an electromagnetic field within the patient to oscillate implanted beacon transponders. When the field is turned off, the transponders emit a signal that is received by the system to determine their positions within the prostate. This function is performed 10 times per second to provide real-time target tracking. There is no use of x-rays and therefore no added radiation exposure for the patient. At the time this study was designed, we believed that real-time tracking offered a more complete solution to the problem of intrafractional prostatic displacements than did punctuated imaging – particularly when very large and few treatment fractions are prescribed as in SABR – and would confidently permit the use of very narrow treatment margins in order to optimize the therapeutic ratio from a geometric perspective. Pre-study testing with beacon-implanted, non-protocol patients supported the selection of the 2-mm action threshold as an acceptable compromise between system sensitivity for triggering a corrective action and throughput efficiency. Of the 510 total SABR fractions delivered to the 102 patients reported here, the 2-mm threshold was observed to be breached at least once during 132 fractions (25.8%), and excursions of >2 mm for >2 min requiring patient and target realignment were observed during 26 fractions (5.1%). Among all treatment fractions, the average time observed between beam-on and beam-off was 14.2 min.

## Conclusion

Favorable 5-year results of this phase II trial support the use of non-robotic, real-time target tracking SABR for low-risk prostate cancer.

## Conflict of Interest Statement

The author declares that the research was conducted in the absence of any commercial or financial relationships that could be construed as a potential conflict of interest.
